# A Novel Matrisomal-Related LncRNA Signature Associated With Survival Outcome and Immune Evasion in Patients With Gastric Cancer

**DOI:** 10.3389/fonc.2022.926404

**Published:** 2022-06-24

**Authors:** Yuan Yang, Li Shi, Jun Zhang, Ya Zheng, Guozhi Wu, Jie Sun, Min Liu, Zhaofeng Chen, Yuping Wang, Rui Ji, Qinghong Guo, Yongning Zhou

**Affiliations:** ^1^ The First Clinical Medical College, Lanzhou University, Lanzhou, China; ^2^ Department of Gastroenterology, The First Hospital of Lanzhou University, Lanzhou, China; ^3^ Gansu Key Laboratory of Gastroenterology, Lanzhou University, Lanzhou, China; ^4^ Digestive Endoscopic Center, The First Hospital of Lanzhou University, Lanzhou, China

**Keywords:** matrisomal pattern, gastric cancer, overall survival (OS), immune regulation, risk model

## Abstract

**Background:**

Different matrisomal patterns are shared across carcinomas. However, little is known about whether there exists a unique tumor matrisome that modulates GC progression and immune regulation.

**Methods:**

We conducted a genome-wide analysis based on matrisomal-related lncRNAs (MRLs) in 375 patients with GC from the Cancer Genome Atlas (TCGA) database. Patients were split into the training set and validation set at a ratio of 1:1 using the R package cart. Pearson correlation analysis (PCA) was performed to identify lncRNAs that correlated with matrisome based on differential expression genes. Subsequently, we performed univariate Cox regression analyses and lasso Cox analysis on these lncRNAs to construct a risk model. Considering the primary effect of GRASLND on the GC prognosis, we chose it for further validation in an experimental setting.

**Results:**

We identified a 15-MRL signature to predict overall survival and immune cell infiltration of patients with GC. The AUC values to predict 5-year outcome in three sets were 0.89, 0.65, and 0.78, respectively. Further analyses suggested that the high-risk group showed more obvious immune cell infiltration, and demonstrated an immunologically “cold” profile. *In vitro*, knockdown of GRASLND could inhibit the invasion capability of GC cells, and downregulate the protein expression of crucial matrisomal-related gene MMP9.

**Conclusions:**

The 15-MRL gene signature might serve as a relatively good predictive tool to manage patients with GC.

## Introduction

Gastric cancer (GC) represents one of the most common digestive malignancies worldwide, particularly in East Asia. The number of deaths was 768,793 in 2020 (1). Despite recent advances in GC treatment, a significant proportion of GC patients are diagnosed at the time of later disease progression (2). The outcomes of GC patients still need to be improved. Chemotherapy has been widely applied as a clinical treatment for advanced GC (3). However, these drugs show few satisfactory therapeutic effects due to chemoresistance or other adverse events (4), which limited their further application in some cases. As a promising innovative therapy, immunotherapy provides an effective strategy for the treatment of GC (5). Seeking effective immune therapeutic indicators for GC remains a unique challenge (6). Therefore, we aimed to establish a novel risk model associated with immune infiltration and survival outcome of GC patients based on large cancer datasets.

The extracellular matrix (ECM) consists of a complex network of cross-linked proteins and has been extensively investigated in recent years. Alterations in the composition, ultrastructure, and mechanical properties of ECM elements could have an impact on the phenotype of the cell, thus participating in the tumorigenesis and development of cancers. Long non-coding RNAs (lncRNAs) exert a variety of biological functions through various gene-regulatory mechanisms (7). Aberrant expression of lncRNAs contribute to the progression of cancers, including GC (8). Several lines of evidence have demonstrated that lncRNAs are mainly involved in the regulation of ECM through several crucial ECM-related regulators. Previous studies revealed that TGFBI, as an important player in the ECM, was negatively regulated by lncRNA H19 *via* the lncRNA H19/miR-675 axis (9, 10). Gu et al. indicated that lncRNA CTD-2589M5.4 could inhibit ovarian cancer progression *via* regulation of ECM remodeling (11). LINC01089 is a novel conserved lncRNA, which could function as the inhibitor of ECM invasion in breast cancer (Sas-12). Moreover, a recent study has also found that the miR-150-related regulatory axis was involved in biological processes that relied on the ECM in hepatocellular carcinoma (13). Therefore, ECM-related lncRNAs might play crucial roles in tumor progression and immune response. Nevertheless, direct lines of evidence about matrisomal-related lncRNAs in GC are still lacking and need further study.

In this study, we conducted a genome-wide analysis about matrisomal-related lncRNAs and developed a new and robust gene signature based on the TCGA database. This new classification could accurately predict survival outcome and was associated with immune infiltration. Furthermore, several experiments *in vitro* showed that knockdown of GRASLND could regulate matrisomal-related gene MMP9. This might provide new insights into exploring the regulatory roles of matrisomal-related lncRNAs in GC.

## Methods

### Data Source

**Figure 1:**
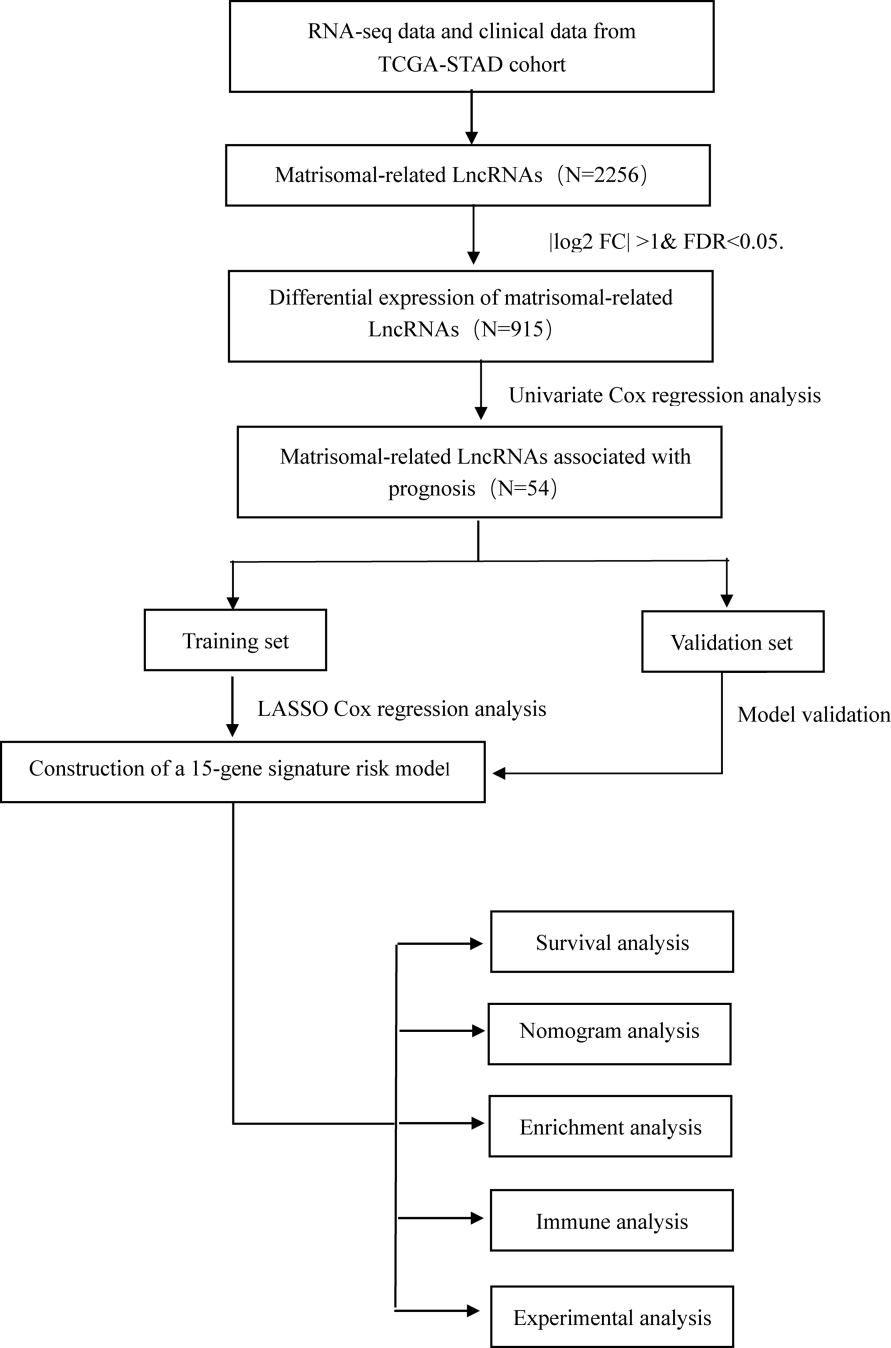
The flowchart of this study.

We downloaded high-throughput sequencing profile data of 375 GC patients from the TCGA public database and removed four samples missing clinical follow-up information. LncRNAs and protein-coding RNAs were annotated based on the GENCODE project (https://www.gencodegenes.org/) (14). A total of 1,068 matrisomal-related genes were obtained from the M.I.T. “Matrisome Project” website (www.matrisomeproject.mit.edu/other-resources/human-matrisome). The workflow is illustrated in **Figure 1**.

### Identification of Matrisomal-Related LncRNAs

The Pearson coefficient was used to investigate links between the potential lncRNAs and the matrisomal-related genes. The absolute value of a correlation coefficient of more than 0.4 and a *p*-value of less than 0.001 were set as threshold values (15–17). The candidate matrisomal-related lncRNAs were considered for subsequent analyses.

### Construction of a Matrisomal-Related LncRNA Risk Model

Differentially expressed matrisomal-related lncRNAs (DE-MRLs) between the cancer and normal tissues were firstly obtained based on R package “limma”. Our criteria were set as |log2 fold change (FC)| > 1 and FDR < 0.05. Based on the survival information for patients with GC, we screened out prognostic matrisomal-related lncRNAs (*p* < 0.001) using univariate Cox regression analysis. Then, GC patients were randomly split into the training and validation set at a ratio of 1:1 using the R package “caret.” We applied lasso analysis in the training set using the R package “glmnet”. A coefficient for each prognostic matrisomal-related lncRNAs was generated accordingly. Multivariate Cox regression analysis was performed to propose the following formula: $\text{Risk Score}=\sum_{i=1}^{n}bi*Si$ . GC patients were classified into high- and low-risk groups using the threshold of median value.

### Evaluation of the Prognostic Signature

Kaplan–Meier survival analysis was employed to investigate overall survival discrepancy between the different risk groups. Subsequently, receiver operating characteristic (ROC), decision curve analysis (DCA), and concordance index (C-index) were used to evaluate the performance of this prognostic model. Additionally, we constructed a nomogram to predict 1-, 3-, and 5-year survival status. Meanwhile, the calibration curve was performed to evaluate the predictive power of the nomogram.

### Immune Cell Infiltration Analysis

Seven immune infiltration prediction algorithms based on RNA-seq were utilized to compare tumor-infiltrating immune cell (TIC) infiltration between the different risk groups. Subsequently, we examined the relationship between the proportions of TICs and candidate matrisomal-related lncRNAs using the CIBERSORT algorithm. Additionally, we performed ssGSEA analysis to quantify the immune regulatory roles. Finally, several common immune checkpoint genes and TIDE score were selected and evaluated between two groups.

### Cell Lines and Transfection

Human GC cell lines, AGS and MKN45, were obtained from the Cell Bank of the Chinese Academy of Science. Two cell lines were cultured in RPMI 1640 medium containing 10% fetal bovine serum (FBS: Gibco). GenePharma (Shanghai) synthesized small interfering RNA (siRNA) specific for GRASLND. siRNA was diluted into 100 μl of Opti-MEM Medium, and then transfected into AGS and MKN45 cells using 100 μl of Opti-MEM Medium with Lipofectamine 3000 (Invitrogen, CA, USA). Knockdown efficiency of the siRNA was assessed using quantitative PCR. The sequence was as follows (18):

GRASLND sense-1 GCUUUGACUUAGACUUCUAGC

GRASLND antisense-1 UAGAAGUCUAAGUCAAAGCUU

GRASLND sense-2 CUGUGAUGGUUAAUGUUAAGU

GRASLND antisense-2 UUAACAUUAACCAUCACAGGG

### Quantitative Real-Time PCR

TransScript One step gDNA kit (TransGen, China) was utilized to achieve complementary DNA (cDNA) synthesis following the manufacturer’s instructions. RT-PCR was performed using TransStart Top Green qPCR SuperMix (TransGen, China). The genetic expression level was normalized to the internal control GAPDH. The standard 2^−ΔΔCt^ method was utilized to analyze the relative lncRNA abundances. Specific primers utilized in this study are as follows (18):

GRASLND Forward AGGATTCAGGGGATGCACAG

GRASLND Reverse TGGGCTGAAGATGAGACGTT

### Invasion Assays

Cell invasion assays were conducted as previously described (19). Matrigel was diluted at a ratio of 1:8 with PBS. After transfection, 1 × 10^5^ cells were added to the upper chamber with 200 μl of serum-free medium. Complete cell culture medium (600 μl) was added to the lower chamber. Then, they were incubated for 48 h at 37°C. Finally, 4% PFA was used to fix all the inserts for 20 min. The inserts were immersed in 0.5% crystal violet for 20 min and quantified using a microscope.

### Western Blot Analysis

The procedures of Western blotting were followed as described in previous reports (20). The primary antibody used was MMP9 (1:1,000, CST). The endogenous protein GAPDH (1:1,000, CST) was utilized for normalization.

## Results

### Identification of Matrisomal-Related LncRNAs in Gastric Cancer

**Figure 2:**
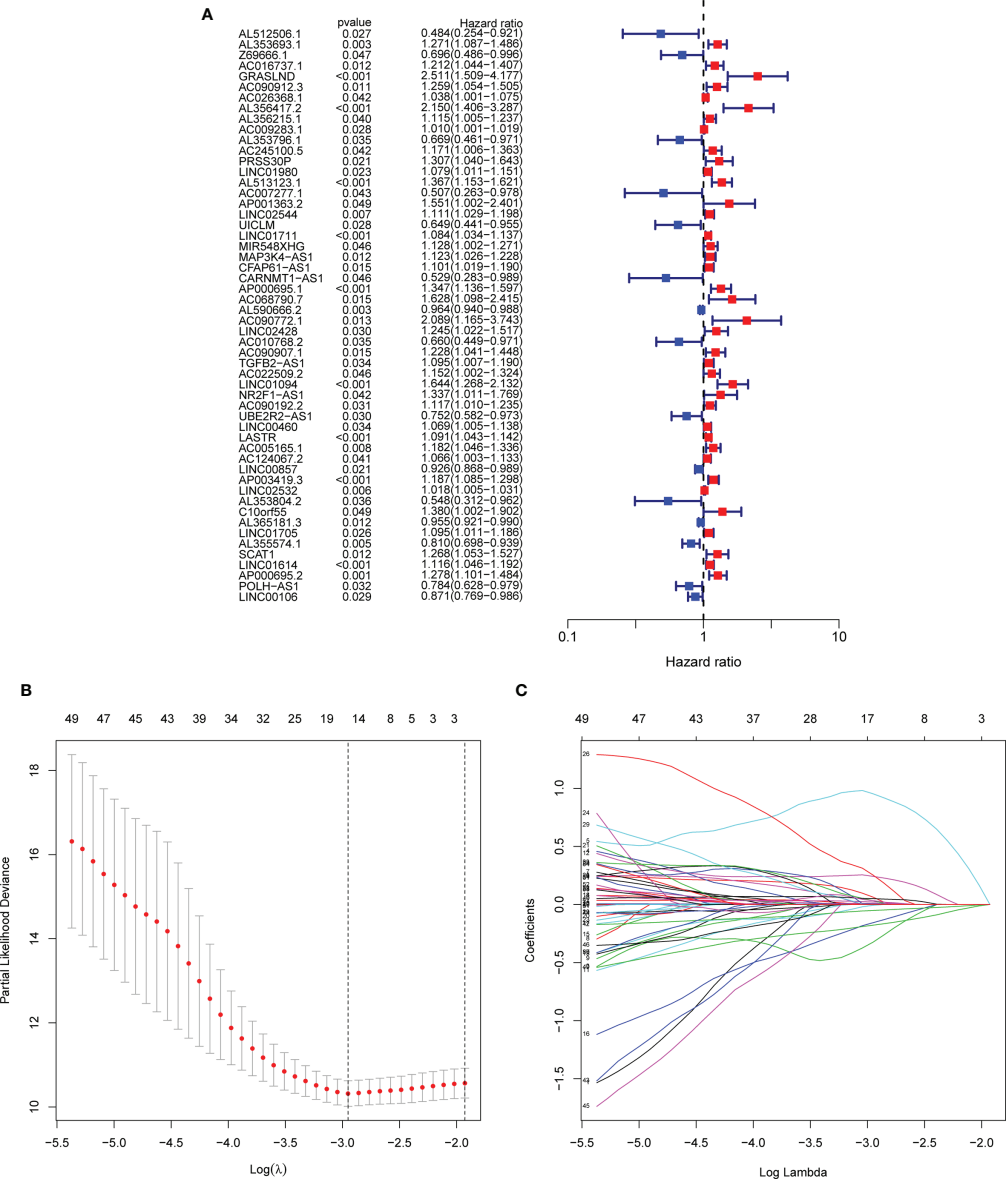
Screening of matrisomal-related lncRNAs and construction of a risk model. **(A)** Forest plot shows the *p*-value and the hazard ratio of 54 matrisomal-related lncRNAs *via* univariate Cox regression analysis. **(B)**. The change trajectory of each independent candidate variable. **(C)**. The optimal model was eventually determined when lambda = 15.

Firstly, we identified 2,256 matrisomal-related lncRNAs (**Table S1**). Subsequently, we carried out differential gene expression analysis between the tumor and normal tissues, and obtained 912 differential expression non-coding RNAs. Finally, 54 lncRNAs were found to be significantly associated with the OS of GC patients using univariate Cox regression analysis (**Figure 2A**).

### Development and Validation of the Matrisomal-Related LncRNA Risk Model

A total of 371 GC patients were randomly segregated into two sets at a ratio of 1:1. Lasso Cox analysis was further performed in the training set, and a coefficient for each prognostic matrisomal-related lncRNA was generated accordingly. A 15 matrisomal-related lncRNAs model was eventually developed using the coefficient value (**Figures 2B, C**). The risk score of each sample was as follows:


$$\begin{array}{l} \text{risk score}=\text{AL}353693.1*0.064+\text{Z}69666.1*\left(-0.325\right)+\text{GRASLND}*0.953+\text{AC}009283.1*\\ 0.004+\text{AC}245100.5*0.242+\text{AC}007277.1*\left(-0.157\right)+\text{LINC}02544*0.0221+\\ \text{AC}068790.7*0.253+\text{AC}022509.2*0.033+\text{UBE}2\text{R}2-\text{AS}1*\left(-0.007\right)+\text{LINC}00460*\\ 0.010+\text{AC}005165.1*0.050+\text{LINC}00857*\left(-0.052\right)+\text{AL}355574.1*\left(-0.178\right)\;+\\ \text{AP}000695.2*\;0.0323 \end{array}$$

**Figure 3:**
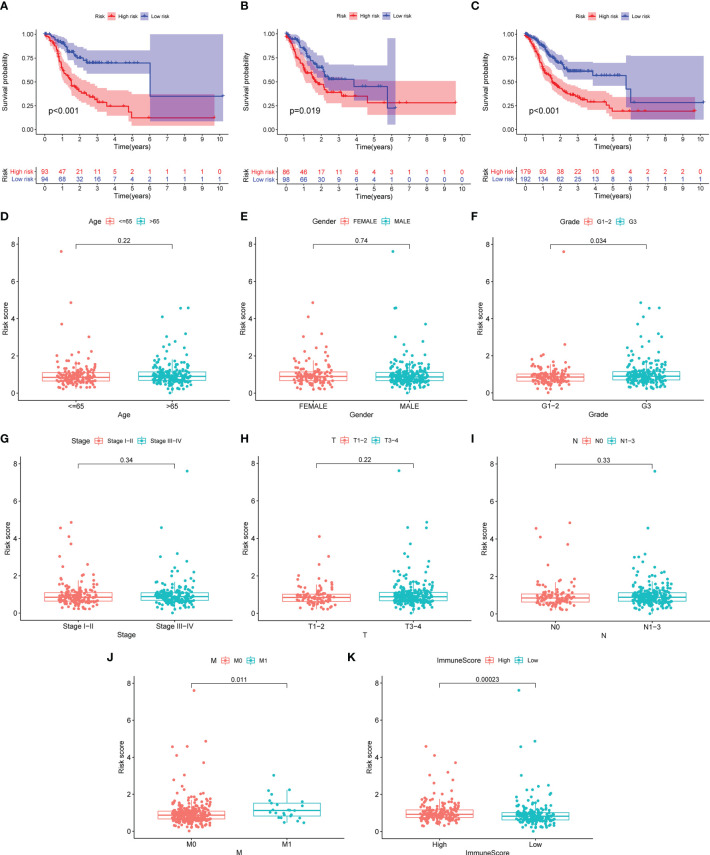
The risk score correlated with overall survival and clinicopathological features. **(A–C)** Kaplan–Meier curves of GC patients between the high- and low-risk group in the training **(A)**, validation **(B)**, and total **(C)** set. **(D–K)** Comparison of risk score distribution between the two groups: age **(D)**, gender **(E)**, G1–2/G3 **(F)**, stage I–II/III–IV **(G)**, T1–2/T3–4 **(H)**, N0/N1–3 **(I)**, M0/M1 **(J)**, and high and low immunescore **(K)**

GC patients were split into two different risk groups based on the median risk value. Kaplan–Meier analysis was employed to investigate OS discrepancy between two groups. As shown in **Figures 3A–C**, GC patients in the high-risk group exhibit significantly shorter median OS (*p* < 0.001). Furthermore, we found that patients with high grade, metastasis, and immune score were associated with higher risk scores, suggesting that there exists a potential relationship between the risk score and the immune status (**Figures 3D–K**).

### Kaplan–Meier Survival Analysis Based on Clinicopathological Characteristics

**Figure 4:**
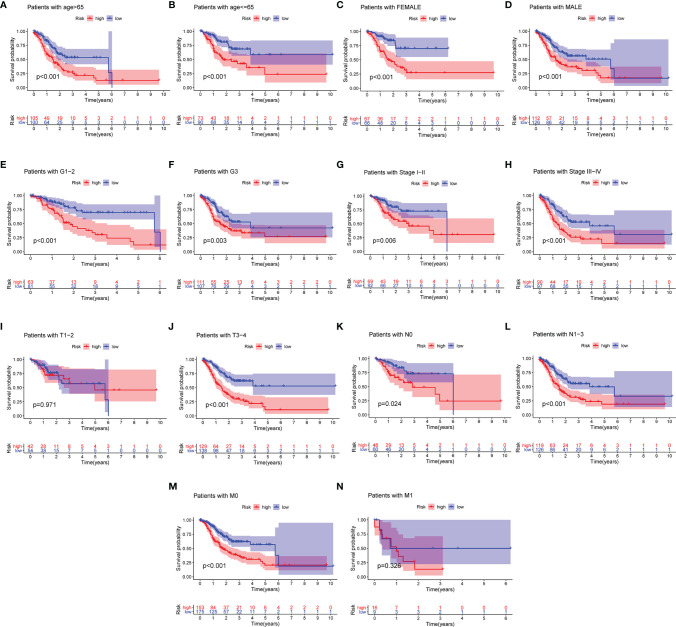
Differential survival outcomes of GC in the clinical subgroup. **(A–N)** Kaplan–Meier curves for age **(A, B)**, gender **(C, D)**, grade **(E, F)**, stage **(G, H)**, T **(I, J)**, N **(K, L)**, and M **(M, N)**.

To assess the prognostic value for established MRL signature, GC patients were split into multiple subgroups based on their clinicopathological characteristics to perform Kaplan–Meier analysis. As shown in **Figures 4A–N**, the results demonstrated that GC patients in the high-risk group were correlated with worse survival in most groups.

### Independent Prognostic Value of the Matrisomal-Related LncRNA Signature

**Figure 5:**
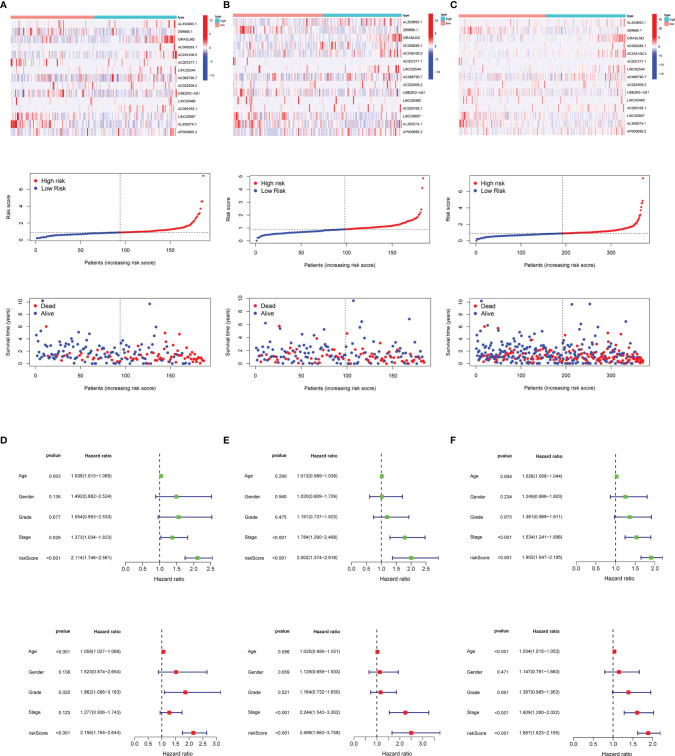
Risk score distribution, survival status, and 15 matrisomal-related lncRNA expression. **(A)** Training set. **(B)** Validation set. **(C)** Total set. Risk score function as an independent prognostic factor. **(D)** Training set. **(E)** Validation set. **(F)** Total set.

Next, we visualized the distribution of risk score, overall survival status, and matrisomal-related lncRNA expression in multiple datasets. We observed that higher risk scores appeared depending on the increasing risk in the training (**Figure 5A**), validation (**Figure 5B**), and total (**Figure 5C**) set. Moreover, the results from two regression analyses indicated that the risk score was closely linked to OS (*p* < 0.001) after adjusting for age, gender, grade, and stage in the training (**Figure 5D**), validation (**Figure 5E**), and total sets (**Figure 5F**), suggesting that the risk score could be used as a potential independent prognostic variable.

### Evaluation of the Validity of the Model

**Figure 6:**
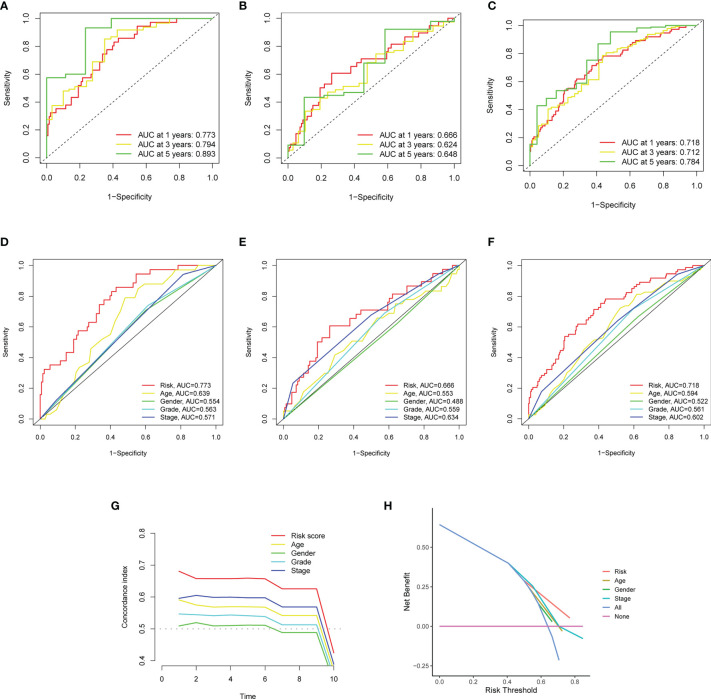
The performance of the matrisomal-related lncRNAs in prognostic prediction. **(A–C)** Time-dependent ROC analysis for evaluating the sensitivity and specificity of this prognostic model in the training **(A)**, validation **(B)**, and total sets **(C)**. **(D–F)** Comparison of clinicopathological features and the risk score in the training **(D)**, validation **(E)**, and total sets **(F)**. **(G, H)** Comparison of clinicopathological features and the risk score in the total sets based on concordance index **(G)** and decision curve analysis **(H)**.

**Figure 7:**
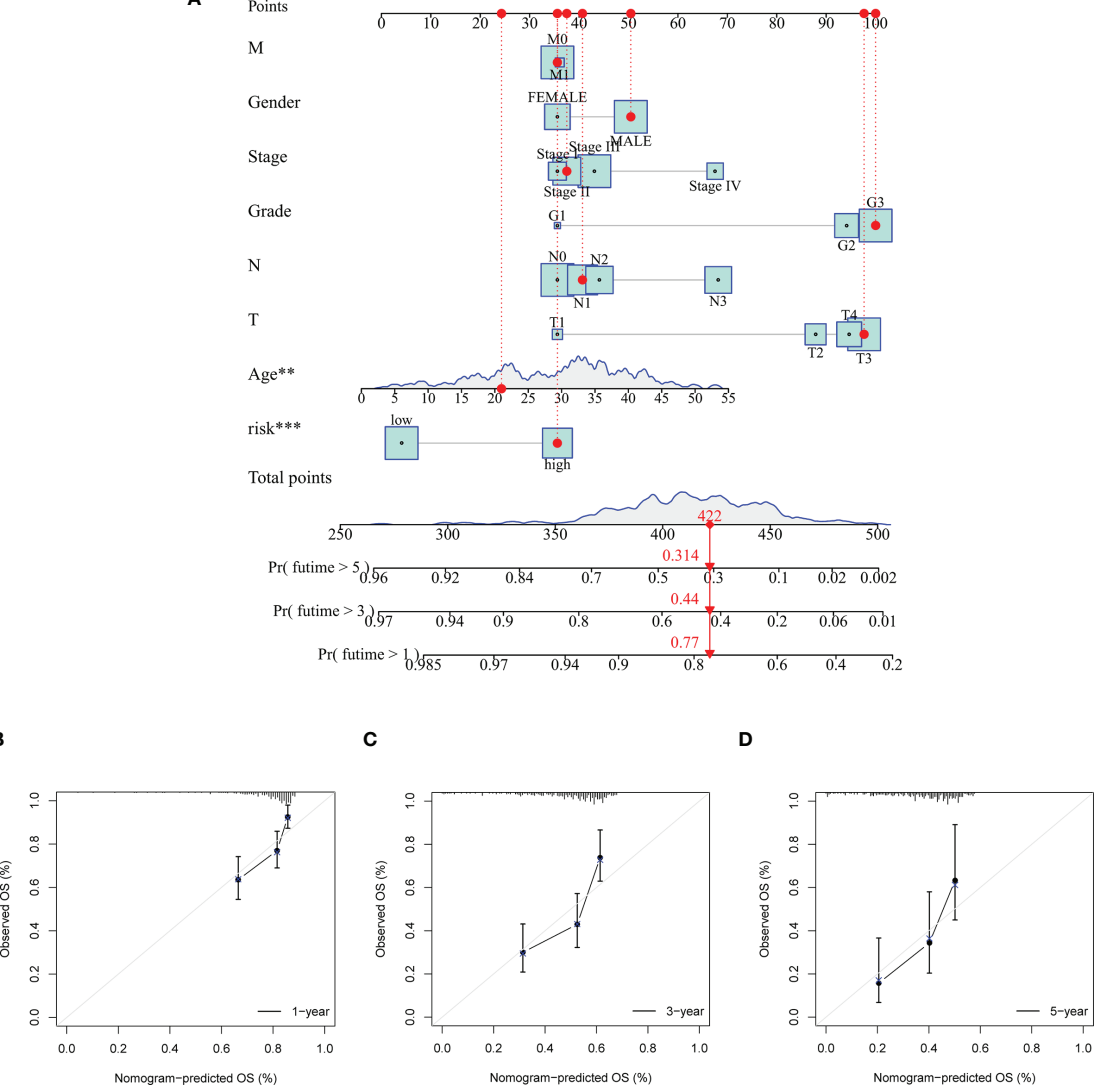
Principal component analysis (PCA) to visualize gene expression patterns in the different groups. The groups defined by all genes **(A)**, matrisomal-related genes **(B)**, matrisomal-related lncRNAs **(C)**, and risk score based on matrisomal-related lncRNAs **(D)**. **p < 0.01, ***p < 0.001

The results in **Figure 6** showed the accuracy of this signature based on time-dependent ROC curves in three sets. The AUC value for 1, 3, and 5 years was 0.773, 0.794, and 0.893 in the training set (**Figure 6A**), 0.666, 0.624, and 0.648 in the validation set (**Figure 6B**), and 0.718, 0.712, and 0.784 (**Figure 6C**) in the total set, respectively. Moreover, we compared the risk score with multiple clinicopathological features (**Figures 6D–F**). The risk score in three sets was more effective than other factors in predicting GC prognosis. Finally, we employed C-index (**Figure 6G**) and DCA (**Figure 6H**) to assess the performance of the risk model. PCA was further performed to reflect the distribution of the overall samples in different statuses (**Figures 7A–D**). The results showed that GC samples were distributed more obviously in distinct directions, indicating a good discrimination for the risk model.

### Construction and Validation of the Nomogram

**Figure 8:**
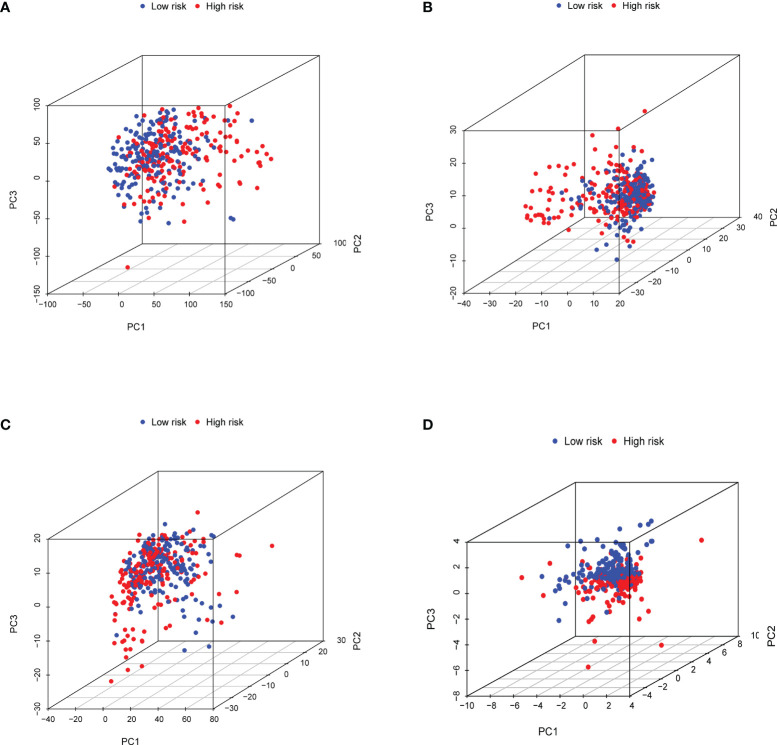
Establishment and validation of a nomogram for predicting the overall survival of GC patients. **(A)** The nomogram was used for predicting the 1-, 3-, and 5-year OS of patients in the total set. Calibration curves of the nomogram predicting OS of 1 year **(B)**, 3 years **(C)**, and 5 years **(D)**.

In order to promote the clinical application of the risk model, the nomogram was eventually built to predict 1-, 3-, and 5-year OS. Several clinical variables, including age, gender, grade, stage, T, N, M, and risk score, were used to establish the nomogram (**Figure 8A**). Moreover, calibration curves were introduced to predict 1-, 3-, and 5-year OS (**Figures 8B–D**). The stability and accuracy of this hybrid nomogram could plausibly function in the areas of GC management.

### Functional Enrichment Analysis of the Risk Signature

**Figure 9:**
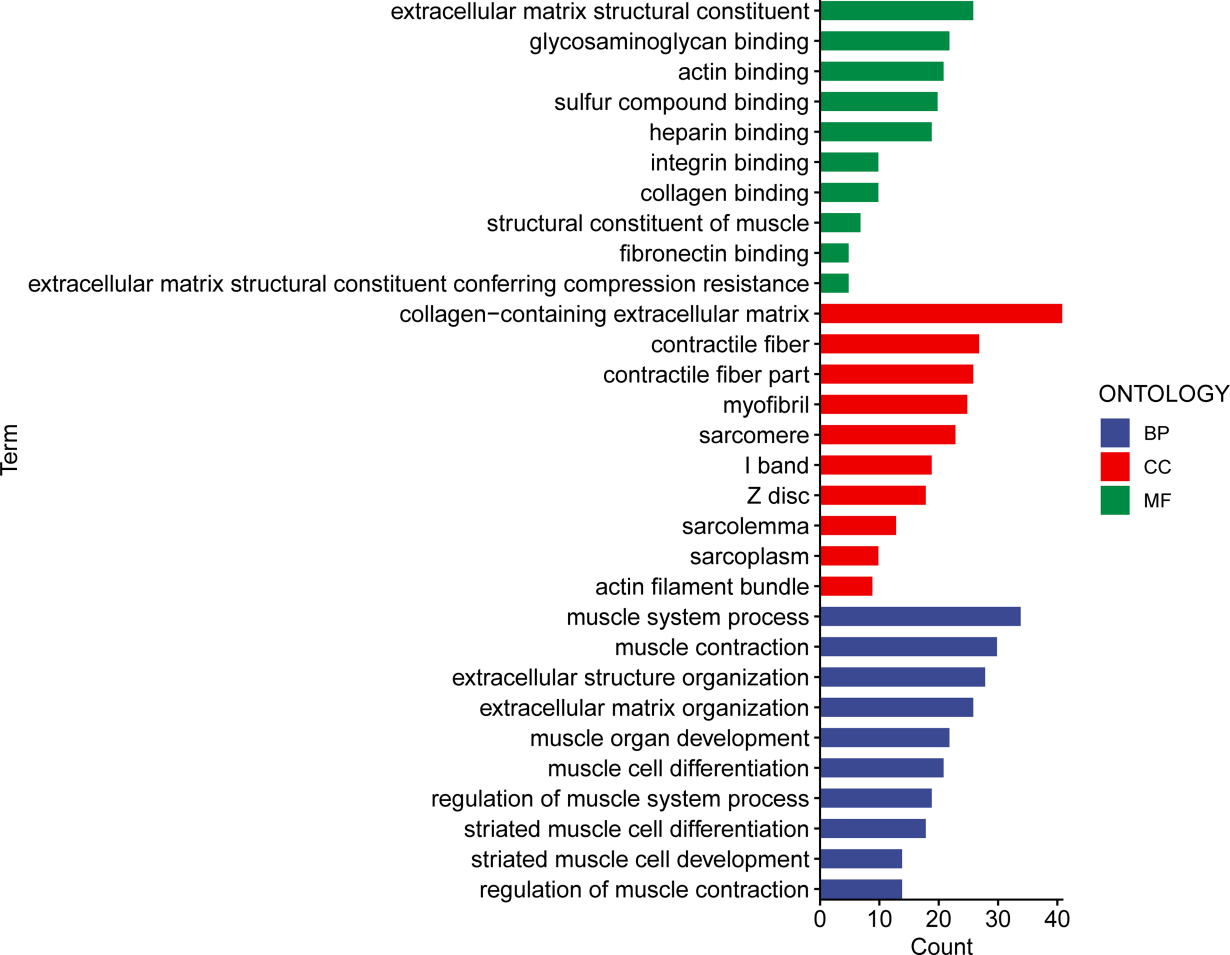
The GO enriched pathways in the different risk subsets obtained from the 15 matrisomal-related lncRNA-based signature.

To determine potential biological roles that were correlated with risk signature, we performed functional enrichment analysis for DEGs between two groups. A total of 241 DEGs were further identified between the low- and high-risk subgroups. Gene Ontology (GO) analysis consisted of cellular component (CC), molecular function (MF), and biological process (BP). The CC GO terms showed that the top 4 enriched pathways were collagen-containing ECM, contractile fiber, contractile fiber part, and myofibril (**Figure 9**). The MF GO terms indicated that the top 4 enriched pathways were ECM structural constituent, glycosaminoglycan binding, heparin binding, and sulfur compound binding (**Figure 9**). The BP GO terms demonstrated that the top 4 enriched pathways were muscle system process, muscle contraction, extracellular structure organization, and ECM organization (**Figure 9**). These results indicated that the risk signature might be functionally implicated in the regulation of ECM networks.

### Functional Enrichment Analysis of the Risk Signature

**Figure 10:**
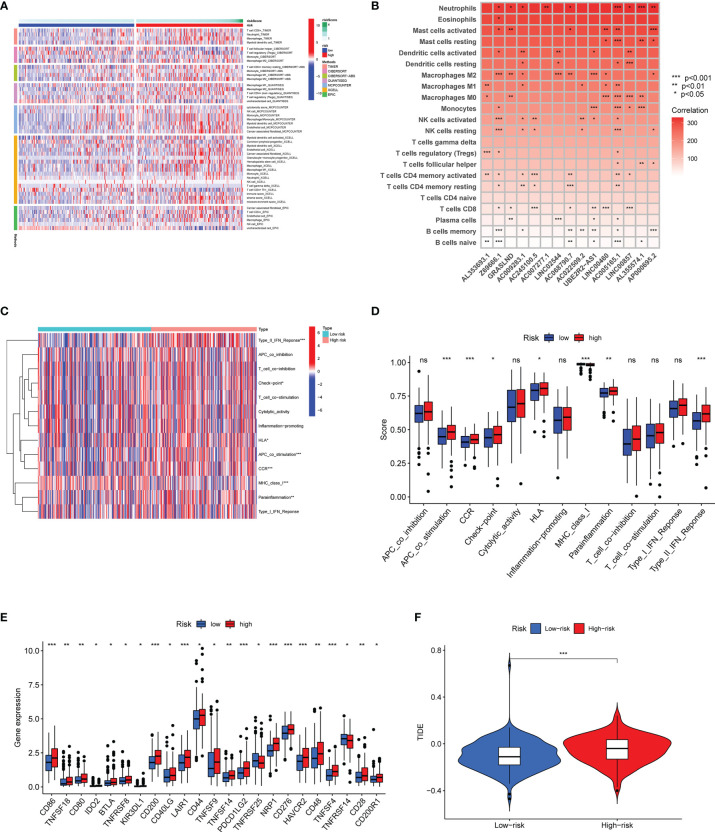
Differential immune features in the high-risk and low-risk subsets. **(A)** The association between infiltrating immune cells and risk scores using seven algorithms. **(B)** The correlation between the expression of 15 matrisomal-related lncRNA-based signature and infiltrating immune cells using the CIBERSORT algorithm. **(C, D)** The heatmap and boxplot show the correlation analysis between immune functions and risk score. **(E)** Box plots revealed the differential expression of the immune checkpoint genes between two groups. **(F)** Correlation of risk score and TIDE score in different risk subgroups. ns no statistical significance, *p < 0.05, **p < 0.01, and ***p < 0.001.

To confirm the association between the risk score and infiltration of immune cells, we conducted further analysis based on seven algorithms (**Figure 10A**). The heatmap was used to display the tumor-infiltrating cell proportions in the different groups. As shown in **Figure 10A**, GC patients in the high-risk group showed higher tumor-infiltrating cell proportions. Macrophage infiltration has been mostly observed in the high-risk group using six algorithms. We further showed the results based on CIBERSORT algorithm as an example, M2 macrophage infiltration was easier to detect in the high-risk group (**Figure 10B**). The results indicated that the proportions of M2 macrophage in the high-risk group might contribute to immune evasion or tumor-promoting effects. Afterwards, we analyzed the potential correlation between the risk score and immune function. As is shown **Figures 10C, D**, the contents of the primary antigen presentation process showed higher proportions in the high-risk group. However, lower MHC_class_I was observed in the high-risk group. The results indicated that this might be associated with the immunosuppressive microenvironment. As is seen in **Figure 10E**, most of the immune checkpoint genes (CD86, CD200, LAIR1, CD44, TNFSF9, PDCD1LG2, NRP1CD276, CD48, and HAVCR2) were dramatically upregulated in the high-risk group. The abnormal expression of genes might contribute to the immunosuppressive microenvironment. To confirm this speculation, we further examined the TIDE score in the different groups. As we expected, our high-risk group showed a higher TIDE score, suggesting that the risk score could discriminate subgroups of GC patients with different immune responses (**Figure 10F**).

### Effects of LncRNA GRASLND on the Regulation of Matrisome in Gastric Cancer

**Figure 11:**
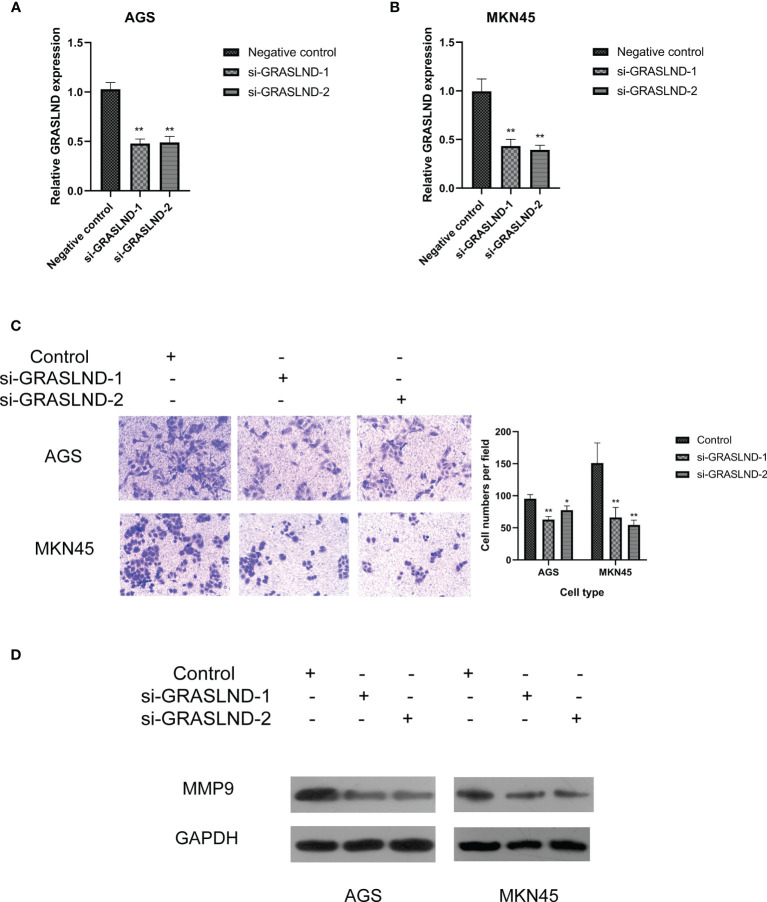
Effects of lncRNA GRASLND on the regulation of extracellular matrix in GC cells. **(A)** AGS and MKN45 cell lines were transfected with si-GRASLND, and qRT-PCR was performed to confirm the knockdown efficiency. **(B)** Knockdown of lncRNA GRASLND decreased the invasive ability of MKN45 cells. **(C)** Knockdown of lncRNA GRASLND decreased the invasive ability of AGS cells. **(D)** Knockdown of lncRNA GRASLND decreased the protein level of MMP9. *p < 0.05, **p < 0.01.

Furthermore, we sought to prove that these lncRNAs in the risk model contribute to the regulation of ECM. Since lncRNA GRASLND showed the highest coefficient and hazard ratio, we chose it to evaluate the potential effects on the regulation of ECM in GC. AGS and MKN45 cell lines were transfected with two si-GRASLNDs, respectively. Subsequently we performed qRT-PCR analysis to confirm the knockdown efficiency (**Figure 11A**). Afterwards, GRASLND depletion significantly decreased the invasive capacity of AGS and MKN45 (**Figures 11B, C**). Furthermore, we analyzed the protein expression of the key enzyme of ECM, MMP9. We found that MMP9 was significantly downregulated after lncRNA GRASLND knockdown in GC (**Figure 11D**). Together, our results suggest that there is a strong correlation between lncRNA GRASLND and the regulation of ECM in GC.

## Discussion

Although surgical resection has been proven to improve the curative effect of GC, the therapeutic effect of advanced and metastatic GC is still not fully satisfactory. Several remarkable advances and breakthroughs have been witnessed in the management of advanced cancers, especially immunotherapy (21, 22). In recent years, researchers have pointed out the dominant roles of tumor heterogeneity in drug resistance and treatment failure (23). Moreover, the complexity of the tumor microenvironment has also exerted a distinct effect on these processes (24, 25). Considering the roles of ECM during tumor progression and immunotherapy resistance, we sought to explore the potential roles of matrisomal-related genes in prognosis prediction and immune regulation.

In this study, we constructed the risk model with a good performance based on matrisomal-related lncRNAs. This model could achieve the robustness of prognosis prediction and assess the robustness of prognosis prediction. Firstly, we obtained 1,068 matrisomal-related genes from the M.I.T. and identified matrisomal-related lncRNAs by Pearson correlation analysis. Among these candidate matrisomal-related lncRNAs, 54 prognostic-related lncRNAs were firstly selected and introduced to the lasso regression. Ultimately, 15 optimal matrisomal-related lncRNAs were obtained for risk model construction. The 1-, 3-, and 5-year AUCs were 0.718, 0.712, and 0.784, respectively, suggesting that the model showed good predictive power for the prognosis of GC patients. The predictive efficiency of the risk model was better than common clinicopathological variables. Multivariate analysis further demonstrated that the risk score was an independent prognostic factor for GC. In addition, to better guide clinical application, a nomogram model was established to predict the prognosis of GC patients. This nomogram model showed a better performance in the prediction of OS. Moreover, we uncovered a positive correlation between risk score and M2 macrophage infiltration. Finally, the experimental validation presented in this study suggested the strong correlation between lncRNA GRASLND and the regulation of ECM in GC.

Recent studies have demonstrated that specific matrix gene sets were predictors of immunosuppression, and thus might predict the efficacy of anti-PD1 therapy (26). Furthermore, Erkan et al. have also identified several stromal signatures that served as predictive and prognostic indicators for patients with pancreatic cancer (12). These results suggested the potential link between matrisome and anti-tumor immunity. Tumor immune-infiltrating cells not only exert an impact on tumor progression, but also induced immune responses to the anti-tumor therapy. Therefore, we explored tumor immune-infiltrating cells to further reveal the underlying mechanisms of immune evasion. In this study, we explored the correlation between risk score and the immune microenvironment. Our results showed that M2 macrophage infiltration was significantly altered in the high-risk group. Several studies demonstrated that M2 macrophage was associated with poor prognosis and disease processes (27, 28). Increasing lines of evidence suggested that different patterns of tumor-associated macrophages (TAMs) played a crucial role in immune escape. M2 macrophages have been proven to be associated with inhibitory cytokine secretion and immune cell infiltration, further attracting negative regulatory factors to the favorable immunosuppressive TME (29, 30). The mechanisms on how M2 macrophages mediated tumor immune escape was complex according to previous studies. Several cells like Th2, Tregs, and MDSCs could achieve M2 macrophage polarization and enhance its infiltration *via* different pathways (31). Conversely, cross-talk among these cells could also lead to a consequent immunosuppressive effect. These findings highlight the crucial roles of M2 macrophage in immune escape. As such, our risk model shows the close relation to M2 macrophage infiltration. Therefore, this model might offer important and unique advantages in the future of cancer immunotherapy.

Subsequently, we studied the interaction between risk score and immune functions. The results of ssGSEA showed that the contents of the antigen presentation process significantly differed in the high-risk group. The above findings further explained the reasons for the tumor-promoting status in the high-risk group and revealed that the immunosuppressive microenvironment exists in this group. These results supported findings from immune cell infiltration analysis. Due to the significance of ICIs, we further assessed the genetic basis of expression of ICI genes, and found the difference between two groups. Interestingly, we found that CD86, CD200, LAIR1, CD44, TNFSF9, PDCD1LG2, NRP1, CD276, CD48, and HAVCR2 were significantly higher in the high-risk group. Finally, the TIDE score was introduced to predict immune response. We found that the high-risk score was associated with a high TIDE score. Collectively, these observations suggested that these patients demonstrated an immunologically “cold” profile not to gain from immunotherapy.

Considering that GRASLND showed the primary effect on the prognosis of GC patients and had a larger coefficient in the risk model, we finally chose GRASLND to conduct functional experiments and explore the correlation with matrisome in GC cells. Our results indicated that knockdown of lncRNA GRASLND could decrease the protein expression of the key enzyme of ECM, MMP9. MMP9 plays a crucial role in the degradation of the ECM (32–34). Moreover, the upregulation of MMP9 was related to metastasis in GC (35, 36). Thus, our experiments confirmed that lncRNA GRASLND was the potential contributor towards regulation of ECM. However, there are still several limitations to our study. External validation of the risk model would be beneficial for its wide use. Hence, clinical samples would also be collected for further verification despite being time-consuming. Furthermore, further experiments are also needed to clarify the unknown functions and the potential mechanism of these lncRNAs.

## Conclusion

In conclusion, we first constructed the 15 matrisomal-related lncRNAs model with a good performance in predicting the prognosis of GC patients. The risk signature might serve as a relatively good predictive tool to manage patients with GC.

## Data Availability Statement

The original contributions presented in the study are included in the article/**Supplementary Material** Further inquiries can be directed to the corresponding authors.

## Author Contributions

Conception and design: YY, LS, and JZ. Administrative support: YNZ and QG. Provision of study materials or patients: YZ, GW, and ML. Collection and assembly of data: JS, ZC, YW, and RJ. Data analysis and interpretation: YY, LS, and JZ. Manuscript writing: All authors. Final approval of manuscript: All authors.

## Funding

This study was supported by the National Natural Science Foundation of China (71964021 and 81960430) and the Natural Science Foundation of Gansu province (21JR1RA117 and 21JR11RA099).

## Conflict of Interest

The authors declare that the research was conducted in the absence of any commercial or financial relationships that could be construed as a potential conflict of interest.

## Publisher’s Note

All claims expressed in this article are solely those of the authors and do not necessarily represent those of their affiliated organizations, or those of the publisher, the editors and the reviewers. Any product that may be evaluated in this article, or claim that may be made by its manufacturer, is not guaranteed or endorsed by the publisher.
